# Supplementary material for: Designed mosaic nanoparticles enhance cross-reactive immune responses in mice
Source: bioRxiv. 2024 Feb 28:2024.02.28.582544. Preprint. [Version 1] doi: 10.1101/2024.02.28.582544 (PMC10925254; doi:10.1101/2024.02.28.582544)
Supplement: Supplement 1 [file media-1.pdf]

Supplementary Information

Designed mosaic nanoparticles enhance cross-reactive immune responses in mice

Eric Wang[1,8], Alexander A. Cohen[2,8], Luis F. Caldera[2,8], Jennifer R. Keeffe[2], Annie V. Rorick[2], Yusuf M. Aida[2,6], Priyanthi N.P. Gnanapragasam[2], Pamela J. Bjorkman[2,*], and Arup K. Chakraborty[1,3-5,7,9,*]

[1]Institute for Medical Engineering and Science, Massachusetts Institute of Technology, Cambridge, MA 02139.

[2]Division of Biology and Biological Engineering[2], California Institute of Technology, Pasadena, CA 91125.

[3]Department of Chemical Engineering, Massachusetts Institute of Technology, Cambridge, MA 02139.

[4]Department of Physics, Massachusetts Institute of Technology, Cambridge, MA 02139.

[5]Department of Chemistry, Massachusetts Institute of Technology, Cambridge, MA 02139.

Present address: [6]School of Clinical Medicine, University of Cambridge, Hills Rd, Cambridge, CB2 0SP, UK

[7]Ragon Institute of Massachusetts General Hospital, Massachusetts Institute of Technology, and Harvard University, Cambridge, MA 02139.

[8]These authors contributed equally.

[9]Lead contact

*Correspondence: arupc@mit.edu; bjorkman@caltech.edu

Table S1. Antibodies in the deep mutational scanning data[1-5] used to calculate escape.

Antibody	RBD epitope class[6]
COV2-2196	class 1
S2H14	class 1
COV2-2165	class 1
COV2-2832	class 1
S2E12	class 1
LY-CoV016	class 1
REGN10933	class 1
C105	class 1
S2X58	class 2
S2D106	class 2
S2H13	class 2
S2H58	class 2
S2X16	class 2
LY-CoV555	class 2
C121	class 2
C144	class 2
C002	class 2
COV2-2096	class 2
COV2-2050	class 2
COV2-2479	class 2
C135	class 3
S2X227	class 3
COV2-2499	class 3
REGN10987	class 3
S309	class 3
COV2-2130	class 3
C110	class 3
CR3022	class 4
COV2-2677	class 4
S2X35	class 4
COV2-2094	class 4
S304	class 4
S2H97	class 4
COV2-2082	class 4
S2X259	class 4

Table S2. The 20 RBD positions with highest escapes from class 1 and 2 anti-RBD antibodies[6] based on DMS data[1-5] and their assigned amino acid. An assigned amino acid was selected as the amino acid with the largest mean escape fraction relative to the WA1 amino acid, which also was not a charged-to-hydrophobic mutation.

Antibody RBD-epitope class	RBD position with high escape	Assigned amino acid
1	417	Y
1	420	Q
1	449	D
1	455	R
1	456	G
1	460	L
1	472	P
1	473	F
1	475	N
1	476	S
1	484	K
1	485	S
1	486	A
1	487	K
1	489	L
1	496	V
1	498	D
1	500	Q
1	501	A
1	504	L
2	346	D
2	447	D
2	449	K
2	450	T
2	452	R
2	455	G
2	456	A
2	472	P
2	473	F
2	475	N
2	481	K
2	483	R
2	484	R
2	485	K
2	486	P
2	487	K
2	489	V
2	490	K
2	493	K
2	494	R

Table S3. Designed RBDs with their specific mutations relative to the SARS-CoV-2 WA1 RBD. 3 mutations are in RBD positions with high escape against class 1 antibodies[6] based on DMS data[1-5] (class 1 escape mutations), and 3 mutations are in RBD positions with high escape against class 2 antibodies (class 2 escape mutations). RBDs that pass experimental validation in terms of expression (Figure 3B) and binding to class 3 and 4 antibodies but not class 1 and 2 antibodies (Figure 3D) are bolded.

RBD Pair	RBD ID	Class 1 escape mutations	Class 2 escape mutations
1	**RBD1**	L455R, F486A, N487K	E484R, Y489V, F490K
	**RBD2**	K417Y, A475N, Q498D	G447D, L452R, Q493K
2	RBD3	L455R, F456G, N487K	E484R, F490K, Q493K
	**RBD4**	A475N, Y489L, N501A	Y449K, L452R, F486P
3	**RBD5**	K417Y, F486A, Q498D	F456A, E484R, S494R
	**RBD6**	L455R, A475N, N487K	I472P, F490K, Q493K
4	**RBD7**	A475N, F486A, N487K	L452R, E484R, Y489V
	RBD8	L455R, F456G, Y473F	G485K, F490K, Q493K
5	**RBD9**	F486A, N487K, Y489L	Y449K, L452R, E484R
	**RBD10**	L455R, I472P, A475N	F456A, F490K, S494R

Table S4. Selected sarbecovirus RBDs along with their GenBank[7] accession numbers, chosen residue numbers based on alignment with the SARS-CoV-2 WA1 RBD, and clade. RBDs that passed experimental validation in terms of expression (Figure 3B) and binding to class 3 and 4 antibodies but not class 1 and 2 antibodies (Figure 3D) are bolded. The clade is defined as described in Starr et al.[8]

Virus	Accession	Residue number	Clade
**LYRa3**	AHX37569.1	310-527	1a
**Khosta-2**	QVN46569.1	307-522	3
**C028**	AAV98001.1	306-523	1a
**SHC014**	QJE50589.1	307-524	1a
**BM48-31**	YP 003858584.1	310-524	3
**BtKY72**	APO40579.1	309-526	3
**pang17**	QIQ54048.1	317-549	1b
RaTG13	QHR63300.2	319-541	1b

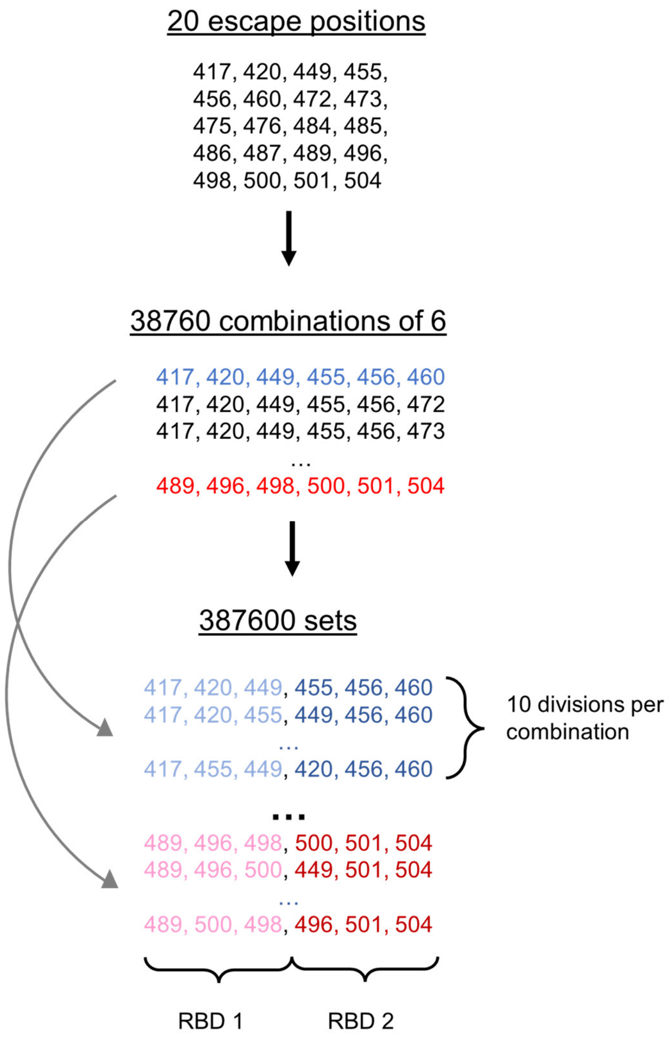

Figure S1. Illustration of how sequences for class 1 escape mutations were generated. From the 20 RBD positions with highest escapes against class 1 anti-RBD antibodies, we generated all 38760 possible combinations of 6 positions. For each combination, we further generated all possible ways to divide the 6 positions into 2 groups of 3, of which there were 10 possible divisions, resulting in 387600 sets of positions. For each set, 1 group of 3 positions was assigned to RBD1 (colored as light blue or pink), and the other group of 3 positions was assigned to RBD2 (colored as dark blue or red). RBD1 and RBD2 would be mutated in their assigned positions to the amino acids listed in Table S2.

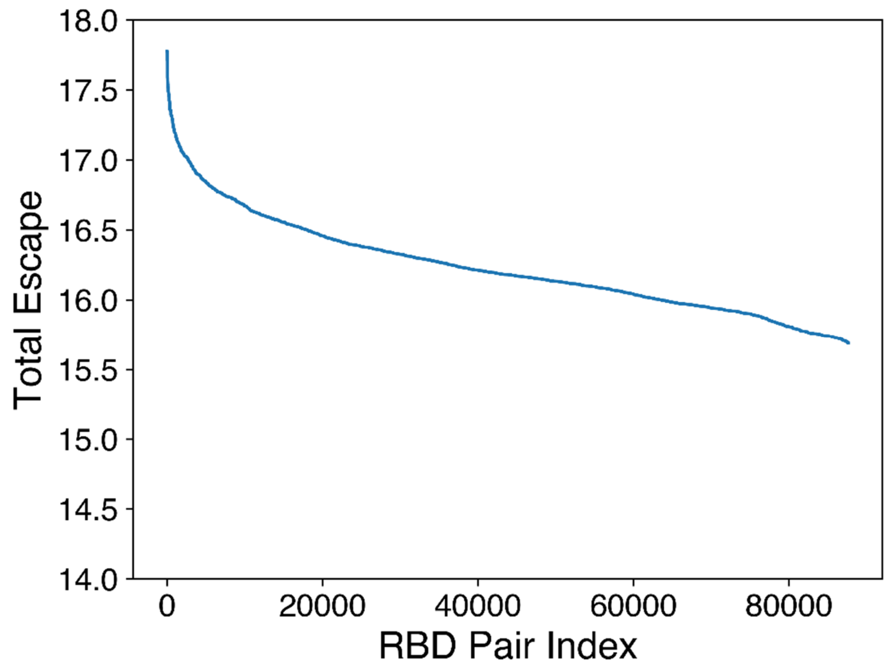

Figure S2. The total escape against class 1 and 2 antibodies for all ~90,000 RBD pairs that pass screening. The total escape is obtained from DMS experiments[1-5] and antibody RBD-epitope classes are defined in Barnes et al.[6]

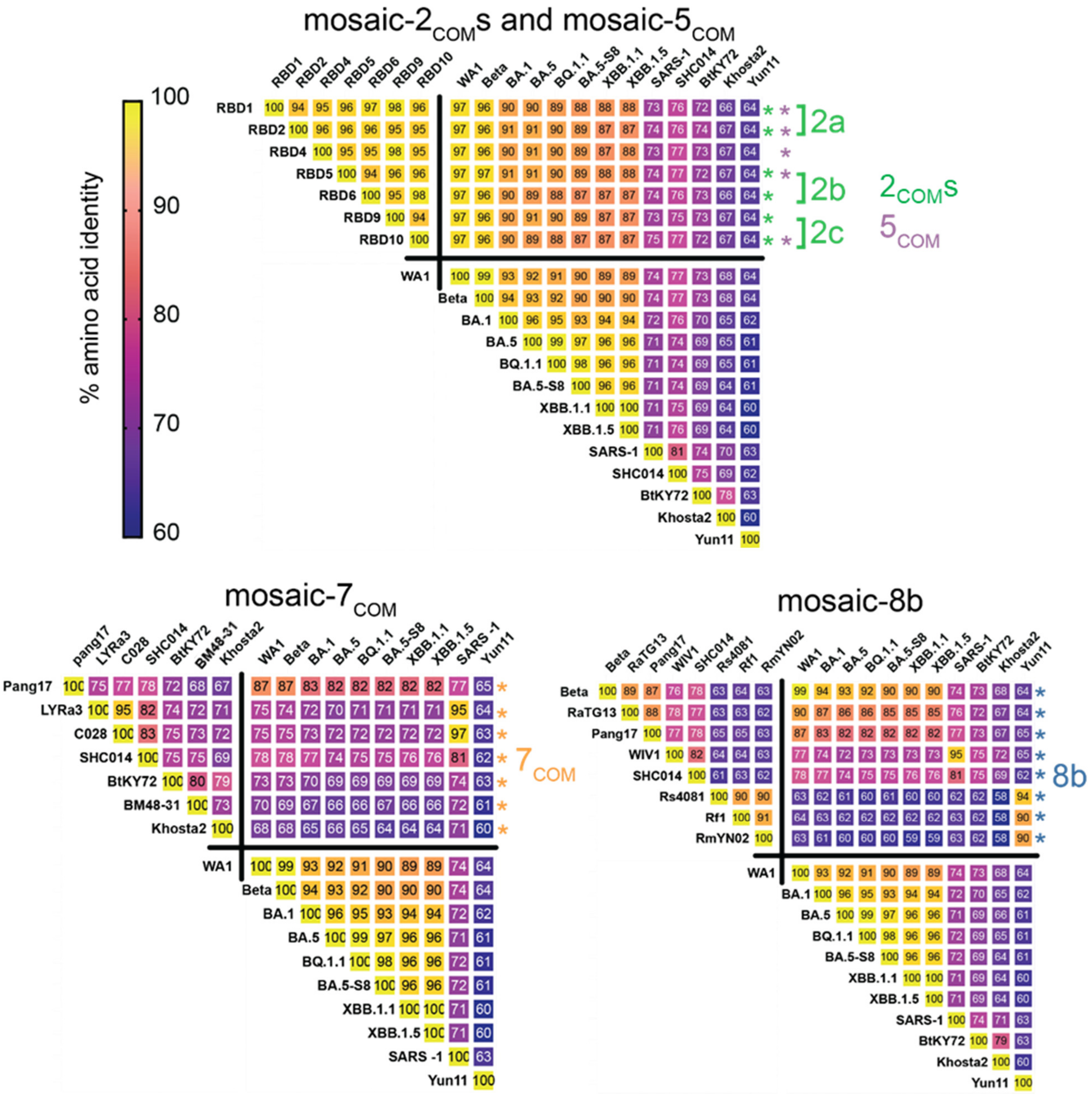

Figure S3. RBD amino acid sequence identities for computationally designed mosaic RBD-NPs and mosaic-8b**.** Asterisks indicate strains used for mosaic-RBD NPs.

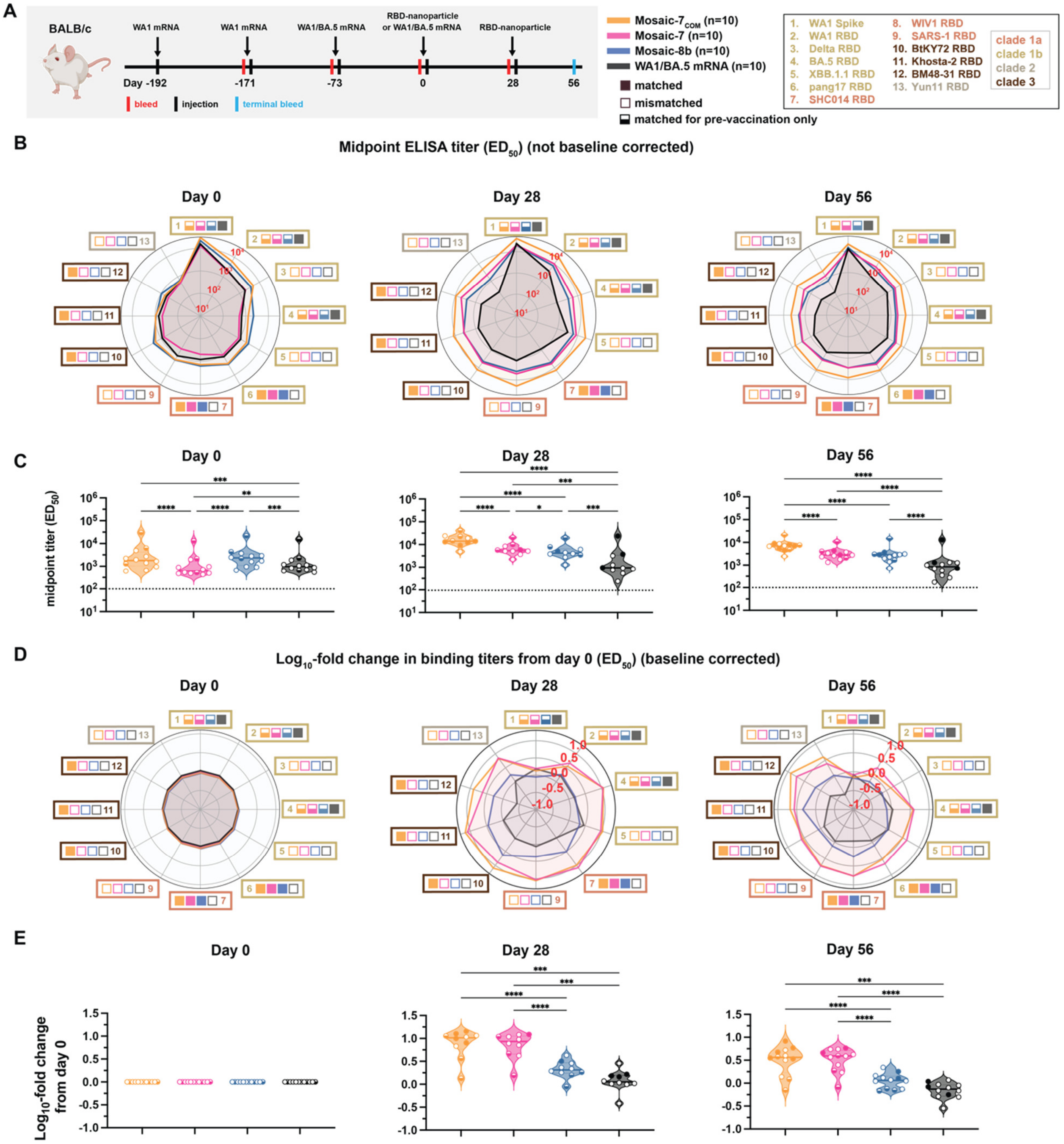

Figure S4. Mosaic-7COM immunization in pre-vaccinated mice elicited superior cross-reactive antibody responses. The mean of mean titers is compared in panels C and E by Tukey's multiple comparison test with the Geisser-Greenhouse correction calculated using GraphPad Prism, with pairings by viral strain. Significant differences between immunized groups linked by horizontal lines are indicated by asterisks: p<0.05 = *, p<0.01 = **, p<0.001 = ***, p<0.0001 = ****.

Binding responses at day 0 (before NP or other vaccine immunizations) showed significant differences across cohorts in titers elicited by the pre-vaccinations.[9] To account

for different mean responses at day 0 between cohorts, we applied baseline corrections in Figure 6 (see Methods). Here, binding data are shown as both baseline corrected (panels B and C) and not baseline corrected (panels D and E).

(A) Left: Schematic of vaccination regimen. Mice were pre-vaccinated with mRNA-LNP encoding WA1 spike and bivalent WA1/BA.5 prior to prime and boost immunizations with RBD-NPs at day 0 and day 28 or an additional WA1/BA.5 mRNA-LNP immunization at day 0. Middle: Colors and symbols (squares) used to identify immunizations (colors) and matched (filled in), mismatched (not filled in), or matched to pre-vaccination (half-filled in) viral strains (squares). Right: numbers and colors used for sarbecovirus strains within clades throughout the figure. (B) ELISA ED_{50} binding titers in serum samples from mice immunized with the indicated immunogens measured at day 0, day 28, and day 56 against spike or RBD proteins from the indicated sarbecovirus strains (numbers and color coding as in panel A). (C) Mean ELISA titers for each type of immunization at the indicated days. Each circle represents the mean ED_{50} titers from mice against a single viral strain of sera from mice that were immunized with the specified immunogen (solid circles=matched; open circles=mismatched; colors for different strains defined in panel A). (D) Mean fold change in ELISA ED_{50} binding titers from day 0 in serum samples from mice immunized with the indicated immunogens measured at day 0, day 28, and day 56 against spike or RBD proteins from the indicated sarbecovirus strains (numbers and color coding as in panel A). (E) Means of fold changes in ELISA titers for each type of immunization at the indicated days. Each circle represents the mean fold change in ED_{50} titers from mice against a single viral strain of sera from mice that were immunized with the specified immunogen (solid circles=matched; open circles=mismatched; colors for different strains defined in panel A).

References

1. Dong, J., Zost, S.J., Greaney, A.J., Starr, T.N., Dingens, A.S., Chen, E.C., Chen, R.E., Case, J.B., Sutton, R.E., Gilchuk, P., et al. (2021). Genetic and structural basis for SARS-CoV-2 variant neutralization by a two-antibody cocktail. Nature Microbiology *6*, 1233-1244. 10.1038/s41564-021-00972-2.
2. Greaney, A.J., Starr, T.N., Gilchuk, P., Zost, S.J., Binshtein, E., Loes, A.N., Hilton, S.K., Huddleston, J., Eguia, R., Crawford, K.H.D., et al. (2021). Complete Mapping of Mutations to the SARS-CoV-2 Spike Receptor-Binding Domain that Escape Antibody Recognition. Cell Host & Microbe *29*, 44-57.e49. 10.1016/j.chom.2020.11.007.
3. Greaney, A.J., Starr, T.N., Barnes, C.O., Weisblum, Y., Schmidt, F., Caskey, M., Gaebler, C., Cho, A., Agudelo, M., Finkin, S., et al. (2021). Mapping mutations to the SARS-CoV-2 RBD that escape binding by different classes of antibodies. Nature Communications *12*, 4196. 10.1038/s41467-021-24435-8.
4. Starr, T.N., Greaney, A.J., Dingens, A.S., and Bloom, J.D. (2021). Complete map of SARS-CoV-2 RBD mutations that escape the monoclonal antibody LY-CoV555 and its cocktail with LY-CoV016. Cell Reports Medicine *2*, 100255. 10.1016/j.xcrm.2021.100255.
5. Starr, T.N., Czudnochowski, N., Liu, Z., Zatta, F., Park, Y.-J., Addetia, A., Pinto, D., Beltramello, M., Hernandez, P., Greaney, A.J., et al. (2021). SARS-CoV-2 RBD antibodies that maximize breadth and resistance to escape. Nature *597*, 97-102. 10.1038/s41586-021-03807-6.
6. Barnes, C.O., Jette, C.A., Abernathy, M.E., Dam, K.-M.A., Esswein, S.R., Gristick, H.B., Malyutin, A.G., Sharaf, N.G., Huey-Tubman, K.E., Lee, Y.E., et al. (2020). SARS-CoV-2 neutralizing antibody structures inform therapeutic strategies. Nature *588*, 682-687. 10.1038/s41586-020-2852-1.
7. Benson, D.A., Cavanaugh, M., Clark, K., Karsch-Mizrachi, I., Lipman, D.J., Ostell, J., and Sayers, E.W. (2013). GenBank. Nucleic Acids Res *41*, D36-42. 10.1093/nar/gks1195.
8. Starr, T.N., Zepeda, S.K., Walls, A.C., Greaney, A.J., Alkhovsky, S., Veesler, D., and Bloom, J.D. (2022). ACE2 binding is an ancestral and evolvable trait of sarbecoviruses. Nature *603*, 913-918. 10.1038/s41586-022-04464-z.
9. Cohen, A.A., Keeffe, J.R., Schiepers, A., Dross, S.E., Greaney, A.J., Rorick, A.V., Gao, H., Gnanapragasam, P.N., Fan, C., Jr., A.P., et al. (2024). Mosaic sarbecovirus vaccination elicits cross-reactive responses in pre-immunized animals. bioRxiv, 2024.2002.2008.576722. 10.1101/2024.02.08.576722.
